# Polyhedral Oligomeric Silsesquioxanes (POSS) for Transparent Coatings: Material Properties and Applications

**DOI:** 10.3390/polym17223050

**Published:** 2025-11-18

**Authors:** Yujia Chen, Zhiwei Bian, Yunhao Wei, Xiaojie He, Xuemin Lu, Qinghua Lu

**Affiliations:** 1 State Key Laboratory of Synergistic Chem-Bio Synthesis, School of Chemistry and Chemical Engineering, Shanghai Jiao Tong University, Shanghai 200240, China; cyj_25@sjtu.edu.cn (Y.C.); bzw0806@sjtu.edu.cn (Z.B.); valentinwei910@sjtu.edu.cn (Y.W.); 2 School of Chemical Science and Engineering, Tongji University, Siping Road No. 1239, Shanghai 200092, China; 2011327@tongji.edu.cn; 3 State Key Laboratory of Micro-Nano Engineering Science, Shanghai Jiao Tong University, 800 Dongchuan Road, Shanghai 200240, China

**Keywords:** POSS (Polyhedral Oligomeric Silsesquioxane), transparent coatings, cage-like structure, functionalization, integration techniques, performance, application

## Abstract

Polyhedral oligomeric silsesquioxanes (POSS) harness their molecularly precise organic–inorganic hybrid cage architecture to deliver hardness, scratch resistance, and programmable functionality for next-generation transparent coatings. Tailoring of solubility, thermal stability, mechanical robustness, electronic characteristics, and interfacial properties is achieved through strategic peripheral modifications enabled by versatile synthetic methodologies—spanning metal catalysis, metal-free routes, and selective bond activation. Advanced integration techniques, including covalent grafting, chemical crosslinking, UV–thermal dual curing, and in situ polymerization, ensure uniform dispersion while optimizing coating–substrate adhesion and network integrity. The resultant coatings exhibit exceptional optical transparency, mechanical durability, tunable electrical performance, thermal endurance, and engineered surface hydrophobicity. These synergistic attributes underpin transformative applications across critical domains: atomic-oxygen-resistant spacecraft shielding, UV-managing agricultural films, flame-retardant architectural claddings, mechanically adaptive foldable displays, and efficiency-enhanced energy devices. Future progress will prioritize sustainable synthesis pathways, emergent asymmetric cage architectures, and multifunctional designs targeting extreme-environment resilience, thereby expanding the frontier of high-performance transparent protective technologies.

## 1. Introduction

Transparent cover coatings serve as critical protective and functional layers in various fields such as photovoltaic modules, display devices, cameras, and other optical components. Transparent overlay coatings play a crucial role as protective and functional layers in numerous fields, including photovoltaic modules, display devices, cameras, and other optical components. The optical transmittance of coatings is a critical performance indicator, influenced by factors such as intrinsic material absorption, scattering losses, and interfacial reflections. Achieving high transmittance requires a low absorption coefficient across the entire visible spectrum and effective suppression of scattering induced by filler aggregation, phase separation, or surface roughness. Conventional nanofillers tend to aggregate into clusters with dimensions exceeding the wavelength of light, which significantly compromises optical clarity [1].

Simultaneously, these coatings must meet diverse requirements, including durability in extreme environments, excellent mechanical properties, and the capability for functional design [2]. However, organic coatings such as polyurethanes are susceptible to UV-induced aging and performance degradation, while inorganic coatings like silica, prepared via the sol–gel process, often fail to meet practical application demands due to their inherent brittleness and propensity for cracking [3,4].

Polyhedral Oligomeric Silsesquioxane (POSS), with its unique molecular-scale organic–inorganic hybrid nanostructure (core general formula (RSiO_1.5_)_n_, commonly *n* = 8, 10, or 12) [5], offers a breakthrough opportunity to address these bottlenecks. The rigid siloxane cage framework of POSS, such as the T_8_ cage (size ~1.0–1.5 nm) and T_12_ cage (up to 1.8–2.2 nm) [6], provides exceptional thermal stability [7,8]. More importantly, the molecularly precise structure of POSS provides an idealized potential for achieving nanoscale dispersion. This stands in sharp contrast to the inherent and difficult-to-overcome aggregation tendency of conventional nanoparticles. In practical applications, sophisticated molecular design strategies, such as constructing covalent networks, are key to suppressing this inherent aggregation tendency and realizing this potential. For instance, Wu et al. successfully prepared highly transparent coatings via this strategy, effectively avoiding light scattering [9]. Crucially, the peripheral organic substituents (R groups, e.g., epoxy, fluoroalkyl, vinyl) of POSS can be precisely modified through various synthetic strategies, including metal-catalyzed and metal-free catalysis [10,11]. This “programmable” functionalization allows for fine-tuning of material properties such as solubility, thermal stability, optoelectronic characteristics, and even mechanical performance [12,13,14,15,16].

Furthermore, the integration of POSS with substrate materials is paramount to the final coating’s performance. Different integration strategies ranging from simple physical blending and covalent grafting to chemical crosslinking [9,17,18], and more advanced techniques like UV–thermal dual curing and in situ polymerization directly influence the coating’s uniformity, interfacial bonding strength, network density, and overall performance [19,20].

This review aims to systematically summarize the research progress and application prospects of POSS-based materials as transparent coatings. The article begins with an analysis of the fundamental structural characteristics of POSS and their structure–property relationships. It then comprehensively examines key fabrication techniques and integration methodologies for POSS-based transparent coatings. Special emphasis is placed on successful application cases that highlight the unique advantages of POSS in advanced technological domains. Additionally, the review systematically evaluates optimization strategies for enhancing the performance of POSS-based transparent coatings, focusing on optical transmittance, mechanical robustness, electronic characteristics, thermal stability, and surface hydrophobicity [9,21,22,23,24,25,26,27,28]. By exploring the multiscale “structure-property-application” relationships in POSS materials, this work aims to establish both theoretical frameworks and practical guidelines for the development of novel, high-performance, multifunctional transparent protective coatings.

## 2. The Basic Characteristics of POSS

### 2.1. Definition and Core Structure

**Figure 1:**
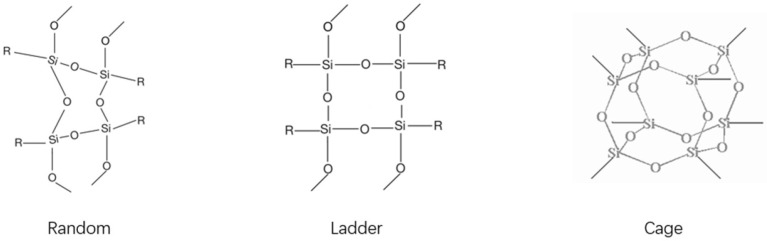
Random, ladder, and cage structures.

POSS is an inorganic-organic hybrid nanomaterial with well-defined cage structures [3,29,30]. Its core formula is (RSiO_1.5_)_2n_ [5], where silicon atoms form polyhedral vertices with tetrahedral substituents (R groups) that dictate key material properties such as solubility, thermal stability, mechanical behavior, electronic characteristics, and surface functionality [31]. Unlike random/ladder configurations, POSS cages (termed *spherosiloxanes*) exhibit a spherical topology due to their three-dimensional ring-connected framework (Figure 1) [31].

### 2.2. Classification and Structural Divergences

**Figure 2:**
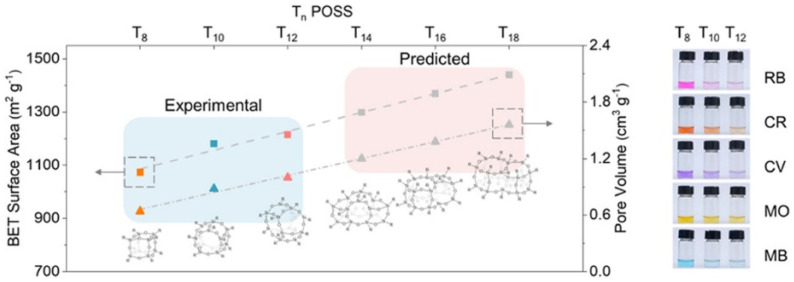
Comparison of the predicted specific surface area (BET) values (red area) and experimental values (blue area) of POSS materials with different structures [6]. Copyright 2023 American Chemical Society.

POSS classification hinges on the number of silicon atoms, where the cage size fundamentally dictates material properties. Increasing cage dimensions from T_8_ to T_12_ linearly enhance porosity and adsorption capacity in POSS-based hybrid polymers (Figure 2) [6], while simultaneously reducing the dielectric constants in low-k systems [32,33]. These macroscopic trends originate from their intrinsic structural differences. Studies have shown that under specific conditions (e.g., in systems with symmetric long alkyl chains), the compact cubic T_8_ cage (such as T_8_C_18_) can promote the formation of dense crystalline packing characterized by parallel-aligned, non-interdigitated alkyl chains, whereas the larger T_10_/T_12_ cages exhibit a more pronounced amorphous character [34]. Consequently, for ammonium-functionalized POSS, the amorphous T_10_/T_12_ fractions demonstrate superior solubility in alcohols compared to their crystalline T_8_ counterparts. This enhanced solubility stems from their disordered nature, which affords looser molecular packing and higher free volume. This structure not only facilitates the penetration of solvent molecules but also requires less energy to disrupt than the tightly packed, crystalline T_8_ framework. This amorphous property, combined with its higher free volume, gives it good application prospects in areas requiring high surface area or controllable pore structure, such as adsorbents and low-k dielectrics [35]. Electronically, DFT studies confirm that the band gap decreases with the cage size increasing, an effect amplified by hydroxyl groups, which redshifts light absorption [36]. Concurrently, gas diffusivity increases with both cage size and the degree of hydroxylation [37], underscoring the strong correlations between the chemical functionality (e.g., overall -OH content) and its transport properties.

## 3. POSS Cage-like Structure: Advantages and Synthesis Methods

POSS, with its unique organic-inorganic hybrid nanocage structure [38,39], features both inherent core stability and customizable peripheral functionalization, enabling synergistic “rigid scaffold–functional modification” design. This design philosophy makes POSS an ideal building block for high-performance transparent cover coatings.

Notably, the POSS cage structure exhibits exceptional stability and structural robustness: linear or cyclic alkyl substitutions typically result in only minimal alterations to the core geometry; core parameters remain largely constant with substituent chain length exerting negligible influence on the overall skeletal framework.

### 3.1. Sol–Gel Method

**Figure 3:**
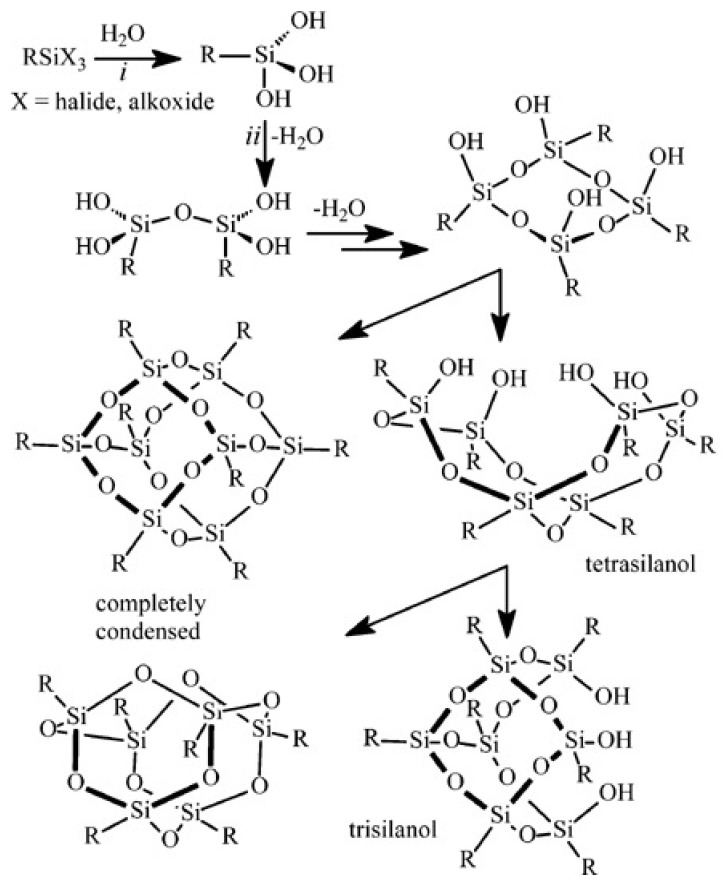
Schematic of POSS synthesis via hydrolytic polycondensation [44]. Copyright © 2004 WILEY-VCH Verlag GmbH & Co. KGaA, Weinheim, Germany.

POSS molecular cages can be synthesized through the hydrolytic condensation of trifunctional silanes, such as organotrichlorosilanes, via a sol–gel process [40]. This involves the following steps: (1) dissolving precursors (metal alkoxides/chlorosilanes) in a suitable solvent, (2) hydrolyzing with water and catalyst, and (3) polycondensation between hydrolyzed products or with unhydrolyzed precursors [41] (Figure 3). Through thiol-ene click chemistry, Laird et al. synthesized a triethoxysilyl-functionalized POSS precursor and subsequently conducted hydrolysis-condensation reactions, thereby incorporating intact POSS cage structures into the hybrid gel network [42]. In recent years, the modified sol–gel method has achieved higher yields. Perry et al. [43] reported the successful synthesis of a model pentapeptide POSS-thioester by utilizing thiol-functionalized polyhedral oligomeric silsesquioxane (POSS-SH) as a soluble carrier. This synthetic process afforded the product in an overall yield of 62% with high purity. This high-yield and high-purity strategy for synthesizing the pentapeptide POSS-thioester provides a viable route for the large-scale chemical synthesis of peptide thioesters, peptides, peptide amides, cyclic peptides, and even proteins.

### 3.2. Corner Sealing Method

The general reaction pathway of the corner-capping method involves the reaction of a partially condensed silsesquioxane with a chlorosilane or alkoxysilane precursor under basic conditions to form a fully cage-closed POSS. This method offers the advantages of high yield and the precise introduction of specific functional groups (e.g., –NH_2_, –SH, –Cl, vinyl), enabling the preparation of T8R7R’-type POSS species (where R’ represents the functional group).

### 3.3. Rearrangement Method

The hydrolysis and condensation of trihalosilanes/trialkoxysilanes or trisilanols typically favor the formation of the T_8_ cage, with larger POSS cages often generated as by-products in low yields. It is noteworthy that, despite sharing an identical chemical composition with the T_8_ cage, these larger POSS structures exhibit significant differences in cage geometry, symmetry, and functionalization characteristics. This uniqueness endows them with superior potential for applications in fields such as photonic crystals, dielectric materials, porous hybrid materials, and functional coatings. Consequently, the investigation and development of synthetic methodologies for large-sized POSS cages are of considerable importance.

Recent studies have revealed that the type of nucleophile plays a decisive role in the structural evolution of POSS cages [45,46]. Furgal et al. [46] employed a fluoride-ion-catalyzed rearrangement of the initial synthesis products [PhSiO_1.5_]_n_ (*n* = 8, 12) and oligomers from PhSi(OEt)_3_ to produce the rare D_5h_ decaphenylsilsesquioxane (SQ) [PhSiO_1.5_]_10_. Their findings support a mechanistic pathway in which the fluoride ion coordinates to and activates a corner of the T_8_ cage, leading to its cleavage. This process involves the stepwise removal of a corner unit as a species such as RSi(OH)_3_, followed by re-insertion to form [RSiO_1.5_]_9_-OH. Subsequently, another corner is inserted to yield [RSiO_1.5_]_10_-(OH)_2_, which finally undergoes condensation to give the closed cage [RSiO_1.5_]_10_ [46].

### 3.4. Characterization Method

Nuclear Magnetic Resonance (NMR) spectroscopy is a routine technique for characterizing synthesized POSS. In addition to ^1^H NMR, which is most commonly used for organic compounds, ^29^Si NMR is particularly suitable for POSS due to their high silicon content. Furthermore, other nuclei can be utilized depending on the functional substituents; for instance, ^19^F NMR is employed when fluorine atoms are present.

Infrared (IR) spectroscopy is another prevalent method for POSS characterization. The asymmetric stretching vibration of the Si-O-Si bond serves as a characteristic signal for the siloxane skeleton. The presence of a strong O-H band typically suggests incomplete cage closure, unless the substituent on the silicon atom is itself a hydroxyl group. Moreover, detecting vibration bands associated with specific chemical bonds in the functional substituents is highly effective for verifying the POSS structure.

## 4. Functionalization Strategies of POSS

POSS properties can be precisely engineered via peripheral structure design, encompassing symmetry, size, and steric effects, chemical nature, and linkage type. POSS properties are significantly influenced by peripheral structure and its symmetry [47,48]. The symmetry of the molecular structure is a consideration in the design of POSS materials. Theoretically, highly symmetric POSS (e.g., octahedral silsesquioxane, T_8_) facilitates nanoscale uniform dispersion through ordered packing, which serves as an ideal structural foundation for achieving mechanical enhancement, such as hardness and scratch resistance [49]. In transparent cover coatings, highly symmetric POSS facilitates the formation of dense and uniform films, reducing light scattering, thereby maintaining or even enhancing the light transmittance of the cover plate. This is critical for the power generation efficiency of photovoltaic modules or architectural daylighting. Deliberate symmetry breaking in POSS molecules enables the creation of Janus particles with unique self-assembly behavior [50,51]. This disruption of symmetry influences the thermal stability, phase transitions, and aggregation properties of POSS-based materials [52]. Asymmetric or Janus-type POSS can be used to design coatings with sophisticated surface functionalities. For instance, one side of the Janus particle can be engineered to be hydrophilic for anti-fogging or underwater applications, while the other side remains hydrophobic for self-cleaning effects, all while maintaining strong bonding to the polymer matrix to enhance coating adhesion. Regarding size and steric effects, the length and bulk of substituent chains directly determine the overall molecular size and steric hindrance of POSS [53]. Short-chain or small-volume groups enhance the reactivity of POSS, but may pose challenges for dispersion due to increased tendency for agglomeration. Conversely, long-chain or bulky groups (e.g., isooctyl, cyclohexyl) significantly improve compatibility with organic polymer matrices, effectively suppress agglomeration [54], and providing excellent plasticization and free volume control. These effects influence material properties such as the glass transition temperature (Tg), gas permeability, and processability [55]. The chemical nature of peripheral functional groups is crucial for POSS functionalization. Introducing diverse substituents onto the silicon atoms of the POSS core can create unique inorganic/organic nanocomposites [56].

### 4.1. The Introduction Method of Functional Groups

#### 4.1.1. Metal Catalysis

Metal catalysis is a widely used functionalization method, employing Rh catalysts for iodoaryl substitution and Pt or Pd catalysts for hydrosilylation reaction [57]. Jagannathan et al. reported Rh_2_(OAc)_4_-catalyzed diazo compound insertion into Si-H bonds to introduce two orthogonal functional groups simultaneously [57]. These two types of groups can serve as orthogonal functional groups for the further derivatization of POSS materials, utilizing reactions such as ester hydrolysis and Suzuki–Miyaura cross-coupling.

For the hydrosilylation of POSS, Walczak et al. [58] evaluated two catalytic systems: the most commonly used Karstedt’s catalyst (Pt_2_(dvs)_3_) and the rhodium(I) chloride dimer ([Rh(cod)Cl]_2_). Both catalysts are commercially available, exhibit high activity and selectivity, and are stable towards air and moisture. The results demonstrated that both catalysts exhibited comparable activity and efficiently afforded the hydrosilylation products. This method enables the preparation of POSS derivatives with well-defined molecular structures and attractive reactivity. By incorporating precisely designed organic substituents at the termini of the silsesquioxane-siloxane chains (Substitution was first performed on the POSS cage structure with siloxane chains, followed by the modification of the chain terminals with functional substituents), this structural feature defines its potential for macromolecular-level applications, holding significant practical value.

Kaźmierczak et al. [59] developed a ruthenium-catalyzed (Ru_3_(CO)_12_) dehydrogenative silylation reaction for the efficient and highly selective coupling of POSS-silanols with hydrosilanes, yielding functionalized silsesquioxanes (SQs) containing a siloxane (Si-O-Si) linkage. The process initiates with a dehydrogenative coupling reaction, catalyzing the formation of an Si-O-Si bond and hydrogen gas from POSS-silanols and hydrosilanes. This is followed by a hydrosilylation reaction, which catalyzes the addition of the intermediate (Si-H-containing SQs) across alkenes (C=C) or imines (C=N). This reaction enables the highly selective formation of the target siloxane products. Notably, it represents the first method to achieve the sequential integration of dehydrogenative coupling and subsequent functionalization reactions in a single pot, without the need for intermediate isolation or additional catalyst.

#### 4.1.2. Metal-Free Catalysis

Janeta et al. successfully synthesized homosubstituted amide-functionalized POSS in a high yield (approximately 95%) using an acyl chloride method [41]. This method proved superior to conventional synthetic approaches employing carboxylic acids or anhydrides, which typically provide yields of only about 60%.

**Figure 4:**
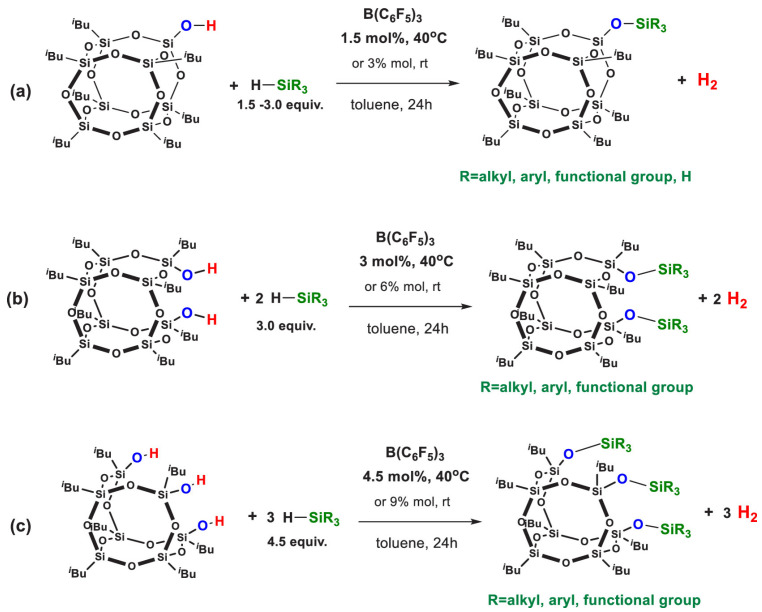
Functionalizations of (**a**) POSS monosilanol, (**b**) POSS disilanol, and (**c**) POSS trisilanol via Dehydrogenative Coupling with Silanes in the Presence of B(C_6_F_5_)_3_ [11]. Copyright 2020 American Chemical Society.

**Figure 5:**
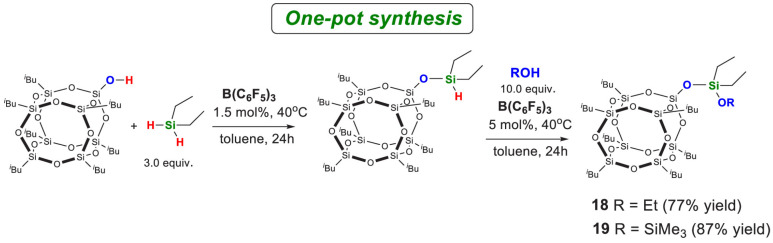
Dehydrogenative coupling of POSS silanols with hydrosilanes catalyzed by B(C_6_F_5_)_3_ and subsequent transformations [11]. Copyright 2020 American Chemical Society.

Kaźmierczak et al. reported B(C_6_F_5_)_3_-catalyzed dehydrogenative coupling of POSS silanols with hydrosilanes for functionalization (Figure 4 and Figure 5) [11]. This method operates under room temperature with readily available catalysts, and generates no corrosive byproducts during the reaction, thereby preventing the retention of harmful substances in the final product.

To further modify POSS derivatives, their substituents can be functionalized. Zhang et al. [60] synthesized the acidic compound POSS-COOH via a click chemistry reaction between octavinyl POSS (POSS-Vinyl) and mercaptopropionic acid, which was subsequently neutralized with three alkali metal hydroxides to obtain POSS-M. Owing to its organic carboxylate groups, POSS-M—featuring a hybrid metal/organic/inorganic structure—significantly enhances compatibility with epoxy resins; due to the inherent characteristics of silicon-containing materials, the composites exhibit excellent thermal stability and flame retardancy; most importantly, alkali metals may catalyze the formation of a stable silicon-containing char layer. Leveraging its unique chemical structure and elemental advantages, POSS-M is expected to expand the categories of silicon-based flame retardants and markedly improve the fire safety of epoxy resins.

#### 4.1.3. Si-O-Si Bond Ring-Opening

**Figure 6:**
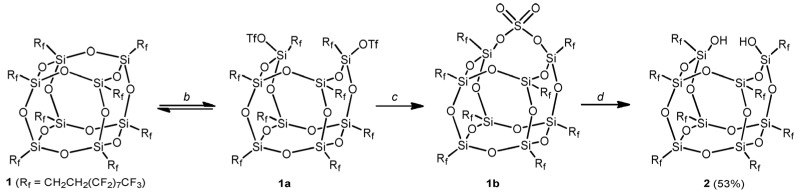
Three-step synthesis converting long-chain fluoroalkyl-substituted closed-cage F-POSS to incompletely condensed silsesquioxanes. All reactions were performed in C_6_F_6_ at 25 °C. Here, b: CF_3_SO_3_H, 75 min; c: NBut_4_HSO_4_, 30 min; d: (CF_3_)_2_CH_2_OH/H_2_O (10:1), 12 h [12]. Copyright 2011 American Chemical Society.

Beyond vertex atom modification, the ring-opening of Si-O-Si bonds within the POSS cage offers another effective functionalization strategy. Ramirez et al. [12,61] developed a three-step method to transform closed-cage F-POSS with long-chain fluoroalkyl substituents into incompletely condensed silsesquioxanes. The cleavage of Si-O-Si bonds generates two hydroxyl sites, facilitating the introduction of new Si atoms with various substituents (Figure 6).

### 4.2. Property Modulation of POSS-Based Materials via Functional Group and Applications in Transparent Cover Plate Coatings

#### 4.2.1. Solubility

**Figure 7:**
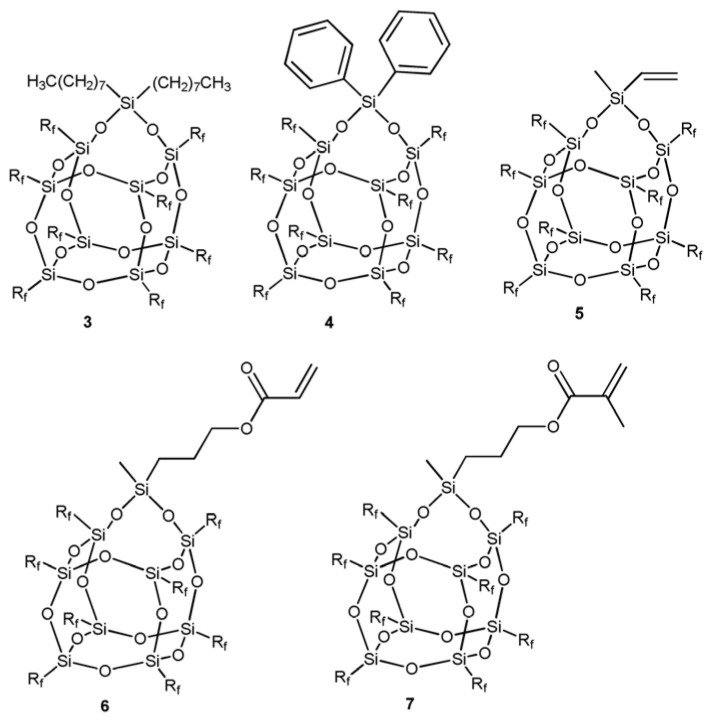
Compounds synthesized from reactive disilanol F-POSS and dichlorosilanes [61]. Copyright 2012 American Chemical Society.

Fluoralkyl polyhedral oligomeric silsesquioxanes (F-POSS) constitute a class of low-surface-energy nanofillers. However, their long-chain fluoroalkyl substituents(R_f_) limit the range of solvents capable of dissolving F-POSS, complicating processing and restricting application prospects [62]. Ramirez et al. [12,61]. developed a three-step synthetic methodology to convert F-POSS into incompletely condensed silsesquioxanes amenable to further functionalization (Figure 7). Functionalized F-POSS derivatives demonstrate enhanced molecular framework stability and markedly altered solubility, which depends on the introduced functional groups. Expanding the range of compatible solvents would significantly enhance the processability of POSS, facilitating reduced processing difficulty and cost, thereby broadening its application scope. However, the practical application value of F-POSS is limited because fluorinated substituents are highly recalcitrant environmental pollutants. The heuristic significance of this study lies in opening the POSS molecular cage for further modification and adjusting the solubility of POSS molecules by varying the types of substituents; this approach can provide valuable inspiration for subsequent research.

#### 4.2.2. Thermal Stability

**Figure 8:**
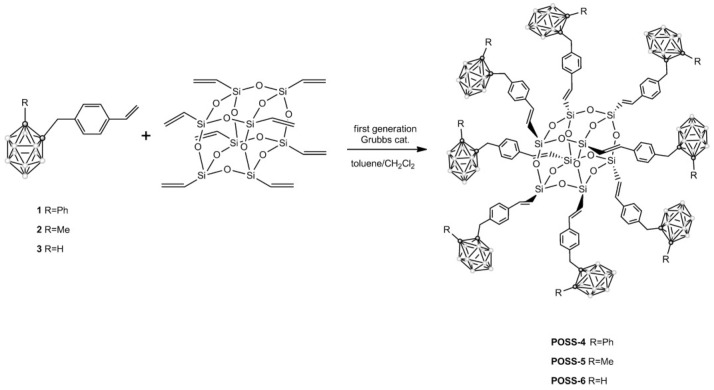
Synthesis of POSS-4, POSS-5, and POSS-6 via cross-metathesis [14]. Copyright 2013 WILEY-VCH Verlag GmbH & Co. KGaA, Weinheim, Germany.

The incorporation of carborane cages into the POSS framework contributes remarkably to the enhancement of thermal stability of POSS molecules. Ferrer-Ugalde et al. [14] synthesized a series of POSS derivatives featuring carboranyl-styrene fragments at every vertex through olefin metathesis. Upon combustion at 1000 °C in air, these carborane-functionalized POSS compounds exhibited mass losses below 10% (Figure 8).

The superior flame retardancy and thermal stability of POSS nanocomposites make them ideal for applications in aerospace, electronics, transportation, construction, and fire-resistant coatings [63]. Research into the thermal stability of POSS-based molecules and POSS-filler transparent cover plate coatings can provide valuable insights for advancing these applications.

#### 4.2.3. Electronic Property

The electronic properties of POSS cage structures exhibit sensitivity to substituents [53,64], enabling the design of materials with tailored properties. Zhen et al. [15] functionalized POSS-T_8_ to modulate HOMO/LUMO frontier orbitals, achieving nanocomposites with tunable bandgaps, charge transport, and exciton binding energies.

Computational results indicate that the internal chemical environment of the POSS molecular cage is primarily maintained by the cage structure itself and is largely unaffected by the functionalization of external organic groups. In contrast, the electronic properties of the overall POSS molecule are significantly influenced by organic group functionalization. For instance, electron-withdrawing groups (e.g., 4-cyanophenyl, Cy) markedly reduce the LUMO energy level while slightly raising the HOMO energy level. Electron-donating groups (e.g., 4-carbazolylphenyl, Car) significantly increase the HOMO energy level. Co-functionalization with both Cy and Car further reduces the band gap. Additionally, the insertion of molecules (e.g., N_2_) within the cage can also substantially lower the LUMO energy level [15].

This work establishes substituent engineering as a precise strategy for tuning POSS electronics, informing high-performance optoelectronic device design. Incorporation of POSS with tailored electronic properties into transparent cover plate coatings enhances optical performance (e.g., refractive index modulation, selective regulation of red/UV absorption and shielding), improves environmental resistance and durability, while enabling anti-static/conductive functionality.

## 5. Integration Techniques for POSS Transparent Cover Coatings

### 5.1. Conventional Integration Methods

**Figure 9:**
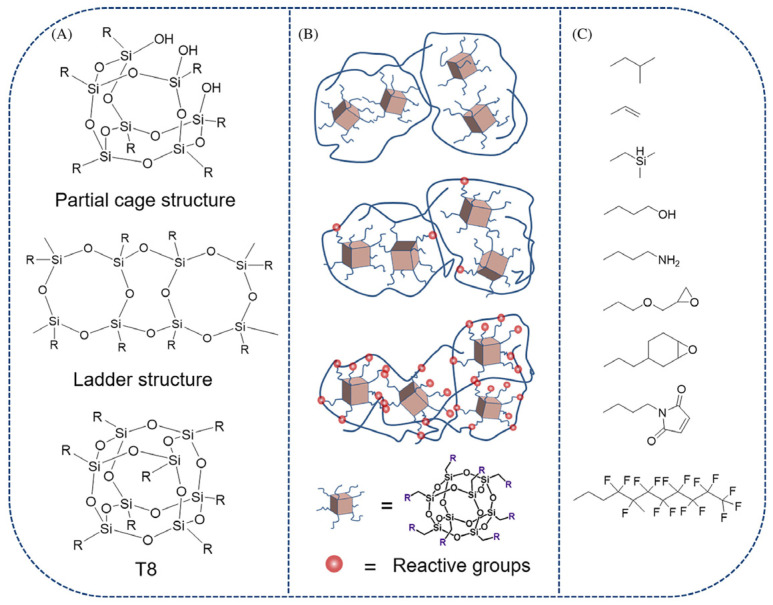
(**A**) Common POSS types; (**B**) POSS-polymer composite structures via physical blending, covalent grafting, chemical crosslinking (The three schematic diagrams in (**B**), from top to bottom, represent physical blending, covalent grafting, chemical crosslinking respectively); (**C**) Typical R substituents in POSS [65]. Copyright 2023 Canadian Society for Chemical.

Conventional POSS integration strategies include physical blending, covalent grafting, and chemical crosslinking (Figure 9) [65].

#### 5.1.1. Physical Blending

Physical blending involves mixing POSS powder solution with pre-synthesized transparent polymers before coating [29]. This straightforward method allows for performance tuning by adjusting the POSS loading [17]. However, simple physical processing methods often lead to particle agglomeration of POSS molecules, resulting in haze and light scattering, which significantly degrade the optical clarity of transparent cover-plate coatings. To ensure stable dispersion of POSS within the coatings, conventional approaches typically employ high-shear mixing, ultrasonication, or ball milling techniques, frequently supplemented with dispersion aids.

#### 5.1.2. Covalent Grafting

Covalent grafting involves tethering POSS moieties to polymer backbones via specific chemical bonds, forming stable architectural hybrids [66], that prevent phase separation while enhancing toughness and thermal stability [18]. However, compared to physical blending, covalent grafting requires stringent control over reaction conditions such as temperature, duration, and catalysts, involves more complex procedures, and incurs significantly higher costs.

#### 5.1.3. Chemical Crosslinking

Chemical crosslinking involves incorporating multifunctional POSS derivatives, such as octa-epoxy POSS and octa-amino POSS, as crosslinkers into polymers. This process enables covalent bonding between polymer chains and the reactive vertex groups on POSS cages, forming dense networks that enhance hardness, strength, abrasion resistance, and thermal/chemical stability, making them ideal for high-performance coatings [9].

### 5.2. Advanced Integration Techniques

#### 5.2.1. UV–Thermal Dual Curing

Beyond conventional methods, incorporating POSS into UV-curable coatings can significantly enhance the mechanical, thermal, and optical properties for transparent cover plate coatings [67,68]. Zhu et al. [68] reported a superhydrophobic coating with applicable light transmittance via a straightforward UV-curing approach. Compared to commercially available UV-cured coatings, this coating demonstrates at least a two-grade improvement in adhesion. Additionally, it exhibits excellent resistance to chemical attacks from acidic (HCl), alkaline (NaOH), and saline (NaCl) solutions.

**Figure 10:**
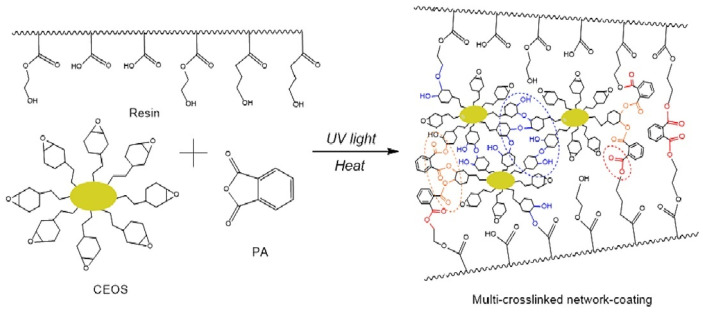
CEOS coating architecture after UV–thermal dual curing [19]. Copyright 2024 Elsevier B.V.

Jae et al. developed a dual-curing process involving initial low-energy UV initiation followed by thermal curing to produce high-hardness films [19]. This method combines cycloaliphatic epoxy-functionalized polyhedral oligomeric silsesquioxane (CEOS) and hydroxylated acrylic resin (AR) to create flexible hard coatings. The CEOS-derived siloxane networks contribute glass-like hardness, while the OH-functionalized AR enhances crack resistance and flexibility. Phthalic anhydride (PA) crosslinks the siloxane-polymer networks through epoxy-anhydride esterification and OH-ester reactions. The sequential UV exposure and thermal curing yield highly transparent films with superior thermal stability (Figure 10).

The core value of a film that simultaneously possesses high thermal stability and high transparency lies in its ability to maintain excellent optical performance and structural integrity over extended periods under harsh thermal conditions, such as during high-temperature processing, continuous operational heating, or abrupt ambient temperature changes. Such films hold broad application prospects in the fields of electronic devices (e.g., display screens, solar cell substrates) and precision optical instruments (e.g., photoelectric sensors).

In recent years, UV-curing techniques have been increasingly adopted for the fabrication of mechanically robust and chemically stable superhydrophobic coatings [57]. The use of functionalized polysiloxane nanoparticles dispersed in photo-curable resins has resulted in abrasion-resistant and transparent coatings that are well-suited for a variety of industrial applications [69,70,71].

#### 5.2.2. In Situ Polymerization

In situ polymerization facilitates the formation of POSS crystallites during synthesis, enhancing dispersion and processability [20]. Meanwhile, in situ-grown POSS layers on substrates enhance adhesion properties and provide atomic oxygen resistance [72]. Xu et al. [73]. fabricated an amine-functionalized POSS layer in situ on polyimide (Kapton) film surfaces using γ-aminopropyltriethoxysilane (APTES), followed by sol–gel deposition of a SiO_2_ coating. During atomic oxygen (AO) exposure tests, the erosion rate of pristine Kapton measured 3.61 × 10^−24^ cm^3^·atom^−1^, whereas the SiO_2_/POSS/Kapton composite exhibited a significantly reduced rate of 0.13 × 10^−24^ cm^3^·atom^−1^, which is only 3.6% of the pristine value, demonstrating exceptional AO resistance.

## 6. The Performance of POSS Transparent Cover Plate Coatings

### 6.1. Light Transmittance

Light transmittance constitutes one of the most critical performance metrics for transparent cover plate coatings [74,75], typically requiring values exceeding 90% in the range of 10–20 nm. Notably, partially condensed POSS exhibits superior dispersion within polymer matrices compared to fully condensed analogues, thereby achieving enhanced transparency films. Incorporating tetrabutyl titanate (TTB)-modified POSS further exhibit exceptional UV-blocking capability while maintaining high visible-light transmittance [21]. This functional synergy confirms the efficacy of POSS/TiO_2_ hybrids as ultraviolet filters, rendering them suitable for sunscreen formulations and horticultural cladding films [76].

Coatings for foldable displays must simultaneously exhibit high hardness, exceptional flexibility, and superior light transmittance. Wu and Xia developed a rigid-yet-flexible coating via photoinitiated free-radical polymerization, achieving high transparency (95.9% at 550 nm), outstanding pencil hardness (9H), and remarkably low modulus (2.53 GPa). Concurrently, incorporation of high-dielectric-constant atomic sulfur increased the dielectric constant of PET film substrates from 3.01 to 3.46. Furthermore, surface modification of the final coating with fluorinated monomer (TFOA) imparted oil and water repellency without compromising other performance characteristics [9]. This study establishes a novel paradigm for developing protective surface coatings for next-generation foldable displays.

Xie et al. [77] synthesized a novel polyhedral oligomeric silsesquioxane (POSS) material with multi-epoxy groups, named EP-POSS, via a click reaction. By selecting a suitable polyamine curing agent, tetraethylenepentamine, and utilizing its efficient curing with EP-POSS, they constructed a multi-point, highly cross-linked network, which enhanced the mechanical strength and chemical resistance of the coating. Furthermore, the introduction of environmentally friendly polydimethylsiloxane imparted flexibility and anti-liquid adhesion properties to the coating. The ultimately fabricated EPOSSPT coating exhibited high transparency, low surface roughness, and high-efficiency repellency against both liquid and solid contaminants, demonstrating excellent self-cleaning and anti-fouling performance. This highly transparent, tough, and flexible coating shows broad application prospects and provides new insights for the design of novel high-performance protective materials.

### 6.2. Mechanical Properties

The silica core of POSS imparts hardness, while its functional groups enhance flexibility, providing dual mechanical benefits on the modified transparent cover plate coatings. Ueda et al. [22] synthesized modified POSS derivatives through an in situ sol–gel reaction in the presence of two alkylsilanes. These POSS fillers not only reduce the refractive index of the PMMA matrix but also improve its thermal and mechanical properties. Mahfuz et al. [78] developed a combined chemical and mechanical processing method to coat carbon fibers with POSS and fabricate laminate composites using vinyl ester resin. Interlaminar shear and low-velocity impact tests showed that the mechanical properties were enhanced by approximately 17–38% compared to control samples without POSS coating.

To investigate the mechanical enhancement effects of siloxanes in polymer composite films, Hwang et al. [23] incorporated a series of structurally diverse silsesquioxanes (SQs, including POSS) into polyurethane acrylate (PUA) polymers. The resulting composite films exhibited exceptional optical transparency and high colorlessness. Furthermore, due to the robust yet flexible nature of the SQ network, the incorporation of all SQ types enhanced the hardness and scratch resistance of PUA films while simultaneously reducing their elastic modulus [23]. These findings offer valuable insights for enhancing the mechanical performance of transparent cover plate coatings.

### 6.3. Electronic Properties

The requirements for the electronic properties of transparent cover plate coatings differ substantially across various applications. For the vast majority of optically transparent cover plate coatings, including camera lens protectors, solar cell cover glass, and standard display surfaces, the coating itself must demonstrate extremely high electrical resistance. This prevents current flow, thereby avoiding leakage currents, short circuits, or interference with internal electronic components.

However, specialized coatings, such as those for touchscreen surfaces, electromagnetic shielding windows, transparent heating elements, and OLED/QLED display encapsulation layers, require moderate-to-high electrical conductivity. Recent studies have demonstrated that the incorporation of POSS along with other conductive materials enables the synthesis of coatings that meet specific requirements, including electrical conductivity, light transmittance, hydrophobicity and other relevant properties.

Nezakati et al. [79] reported a conductive polymer utilizing POSS nanocages as cross-linking units. In this work, using stable dimethylacetamide (DMAc) as solvent, the electrically insulating POSS-PCL matrix (conductivity ≈ 10^−13^ S/cm) transforms into a conductive hybrid nanocomposite upon incorporation of 5 wt.% graphene, achieving the percolation threshold where electrical conductivity exhibits a dramatic surge to 10^−4^ S/cm.

Additionally, Huang et al. [24] fabricated an optimized dielectric thermally conductive epoxy nanocomposite using POSS-functionalized boron nitride nanotubes (BNNTs) as fillers, which exhibited a dielectric constant of 3.6 at 100 Hz (compared to 4.1 for pure epoxy), with dielectric loss reduced by one order of magnitude below 100 Hz at 30 wt% BNNT loading. Simultaneously, they achieve a thermal conductivity enhancement of 1360% (2.77 W·m^−1^·K^−1^ vs. 0.2 W·m^−1^·K^−1^ for epoxy) and significantly reduced coefficient of thermal expansion.

Huang et al. [80] address the critical carrier imbalance in perovskite LEDs by interfacial engineering with insulating POSS. In electrical management, POSS serves as a hole-blocking layer—its deep HOMO level (−6.7 eV) creates an energy barrier between perovskite (−5.9 eV) and TPBi (−6.2 eV), suppressing hole leakage and confining electron-hole recombination within the emissive layer, thereby boosting EQE (External Quantum Efficiency) from 0.02% to 0.35% (a 17-fold enhancement). In conductivity optimization, POSS passivates perovskite NC surfaces, reducing agglomeration of insulating ligands and improving film coverage, which lowers the driving voltage from 6.5 V to 5.8 V and elevates peak luminance to 2983 cd/m^2^ (8× higher than the reference device’s 376 cd/m^2^).

### 6.4. Thermal Stability

POSS has been shown to significantly improve the thermal stability of both transparent coatings and polymers. Wu et al. [25] reported a pioneering approach by synthesizing a liquid POSS (The POSS cage was connected to the triallylsilane group via a thioether linkage), ingeniously achieving ‘molecular-level synergy’ between POSS and carbosilane. This strategy not only resolves the processability challenges associated with solid POSS but also synergistically enhances the thermal stability and overall performance of the coatings. Tanaka et al. [16] investigated the structure-property relationships between the filler architecture and the thermomechanical properties of polymer composites utilizing POSS as a filler. The study revealed that the chemical structure of the POSS side chains is crucial to their enhancing effects; specifically, longer alkyl chains and unsaturated bonds both contribute to enhanced thermal stability and elasticity of the polymer matrix. The authors attributed these enhancements to strong hydrophobic interactions and the potential for chain tangling (for longer alkyl chains), as well as stronger chemical interactions such as π-π stacking (for unsaturated/aromatic groups), which collectively restrict polymer chain mobility and reinforce the composite network. Among all types of POSS studied, the phenyl-functionalized POSS demonstrated the most exceptional overall capability for improving the thermomechanical properties of the conventional polymers most effectively. Kozuka et al. [26] demonstrated that cyclopentyl-substituted POSS enhances the thermal stability of structural color in modified high-molecular-weight block copolymers (BCPs) compared to their isobutyl analogues. This improvement was attributed to the thermally reinforced POSS domains in the cyclopentyl-substituted system. Paajanen et al. [81] improved the thermal stability of polypropylene (PP)-based porous electromechanical films by incorporating cycloolefin copolymer (COC), which has superior thermal resistance, and POSS into the composite.

### 6.5. Hydrophobicity

Transparent coatings with superwetting characteristics (superhydrophilicity or superhydrophobicity) possess broad application prospects. Zhu et al. [82] fabricated a superhydrophilic transparent coating (denoted as the I-coating) using POSS modified with tetrathiol-terminated tetra-poly(ethylene glycol) monomethacrylate (POSS-(SH)4-(PEGMA)4), and simultaneously prepared a superhydrophobic transparent coating (denoted as the O-coating) using POSS modified with thiol and heptafluorinated alkyl acrylate (POSS-SH-(DFMA)7). They systematically compared the similarities and differences between the superhydrophobic and superhydrophilic coatings in terms of anti-fogging, stain resistance, self-cleaning, and anti-biofouling applications. This study can provide a reference for further in-depth exploration of the comparative advantages and disadvantages of superhydrophilic and superhydrophobic technologies, as well as their specific applications.

Marcinkowska et al. [27] modified UV-curable acrylate-based coatings via copolymerization of base resin with polyhedral oligomeric silsesquioxane(4M4F-POSS, a single-molecule compound bearing four methacryloxy and four fluoroalkyl substituents) enhancing the coating’s scratch resistance and hydrophobicity. Ganesh et al. [83] prepared fluoroPOSS-PVDF-HFP nanocomposite blends by mixing two fluorinated polyhedral oligomeric silsesquioxanes (FP8 and FPSi8) separately with poly(vinylidene fluoride-co-hexafluoropropylene) (PVDF-HFP) solution. Transparent superhydrophobic coatings were subsequently fabricated on glass substrates using electrospinning technology [83]. Park et al. engineered highly transparent hydrophobic coatings on plastic substrates through a two-step process: dip-coating (applying polydimethylsiloxane-octadecylamine, PDMS-ODA) followed by hot-pressing (embedding (1H,1H,2H,2H-heptadecafluorodecyl-1-yl)phosphonic acid-modified alumina nanoparticles) [28].

Anti-icing glass is particularly important for application scenarios where ice formation may pose safety hazards or impair functionality. The challenge in anti-icing modification of glass lies in maintaining hydrophobicity while simultaneously addressing the issues of transparency and durability. Zhang et al. [84] developed a highly transparent coating using glycidyloxypropyl-functionalized polyhedral oligomeric silsesquioxane (GPOSS) as a precursor, modified with acrylic acid and perfluorooctyl acrylate. This coating exhibits strong adhesion to glass substrates and effective liquid repellency. The core innovation of the fluorinated GPOSS-based coating developed in this study lies in its inorganic/organic composite structure, which simultaneously achieves high transparency, mechanical durability, and enhanced anti-icing performance.

Furthermore, in certain applications, coatings with high hydrophilicity are also required. Zhu et al. [85] developed a highly hydrophilic coating on transparent polycarbonate substrates using a UV-curing process, achieving a water contact angle of less than 10°. The key hydrophilic functionality of this coating originates from poly(ethylene glycol) methyl ether methacrylate-modified cage-structured polyhedral oligomeric silsesquioxane (PEGMA-modified POSS). The developed hydrophilic coating demonstrated excellent anti-fogging and anti-frosting properties, along with remarkable self-cleaning capability. These characteristics make it a promising material for applications involving transparent optical polycarbonate materials that require enhanced anti-fogging, anti-frosting, and self-cleaning performance (Table 1 and Table 2).

## 7. Application of POSS in Transparent Coatings

With their outstanding comprehensive performance, the POSS transparent cover plate coatings hold broad application prospects in several key fields [2].

### 7.1. Space Environment Applications

POSS coatings are ideal protective materials for the transparent components of spacecraft, especially in the harsh conditions of low Earth orbit (LEO). Xu et al. [73] fabricated a transition layer-enhanced coating. They utilized amine-functionalized POSS derived from APTES(3-Aminopropyltriethoxysilane) as an interfacial transition layer between Kapton and a SiO_2_ coating, constructing a SiO_2_/POSS/Kapton composite structure via the sol-gel method. This coating reduced the atomic oxygen (AO) erosion rate to 3.6% of that of pristine Kapton (0.13 × 10^−24^ cm^3^/atom vs. 3.61 × 10^−24^ cm^3^/atom). This improvement is attributed to the POSS reinforcing the interfacial adhesion and suppressing coating delamination. Beyond coatings, POSS has been successfully integrated into polyimide matrices, leading to practical aerospace materials like main-chain and side-chain POSS-Kapton (MC/SC-POSS-Kapton) and the commercial CORIN^®^ film, which offers high transparency and superior AO resistance. Recent developments focus on optimizing these systems; for example, Wang et al. [87] enhanced the glass transition temperature (Tg up to 297 °C) and dimensional stability of a CORIN^®^-like polymer by incorporating a rigid dianhydride (6FCDA), while maintaining excellent AO resistance (erosion yield as low as 0.17 × 10^−24^ cm^3^/atom).

Lei et al. [88] synthesized copolymerized POSS-polyimide films by copolymerizing a diamine-functionalized POSS (POSS-diamine) with imide monomers, developing POSS-PI composite films. At a POSS loading of 29.7%, the minimum erosion rate was achieved (0.9 × 10^−25^ cm^3^/atom). The significant enhancement in space durability is ascribed to the in situ formed silica passivation layer on the surface. Atar et al. [89] prepared nanocomposite films. The CNT(carbon nano tube)-POSS-PI film exhibited an erosion rate of only 4.8 × 10^−25^ cm^3^/atom under an AO fluence of 2.3 × 10^−20^ atoms/cm^2^, representing a one-order-of-magnitude reduction compared to pristine PI.

Additionally, advanced self-healing capabilities have been developed, enabling in-orbit repair of mechanical damage. These autonomous self-healing, AO-resistant coatings are based on 2-ureido-4[1H]-pyrimidinone (UPy)-functionalized polyhedral oligomeric silsesquioxane (UPy-POSS). This material spontaneously assembles into hydrogen-bonded, three-dimensional supramolecular polymers. Remarkably, UPy-POSS coatings can rapidly autonomously heal mechanical damage—such as cracks—either at 80 °C or directly within the LEO environment, effectively restoring their original atomic oxygen resistance [90].

**Figure 11:**
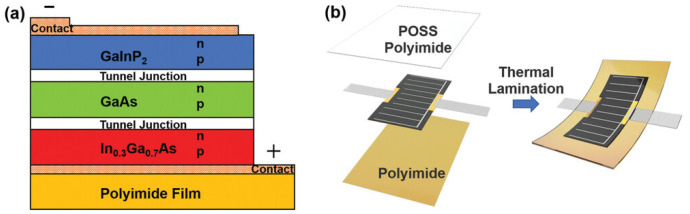
(**a**) Structure of flexible triple-junction GaAs thin-film solar cell. (**b**) Structure of POSS polyimide sealed flexible triple-junction GaAs thin-film solar cell [91]. Copyright 2021 Wiley-VCH GmbH.

Furthermore, their outstanding radiation and ultraviolet resistance make them an ideal choice for the solar cell coating in space. The transparent POSS-polyimide (POSS-PI) film serves as the encapsulation layer for flexible triple-junction GaAs solar cells, successfully overcoming the drawbacks of traditional rigid glass covers, such as high weight, inflexibility, bulkiness, and integration difficulty. Through a thermal lamination process, the encapsulated cells achieve a high photoelectric conversion efficiency of 28.44% (AM0, 25 °C) and demonstrate stable performance upon exposure to AO (>4.1 × 10^21^ atoms cm^−2^) and ultraviolet radiation (>89.5 ESH) (Figure 11) [91].

Although POSS coatings exhibit excellent performance, their practical aerospace applications still face significant challenges. The complex synthesis processes and high costs constrain their production feasibility for large-scale spacecraft, such as commercial satellite constellations. Furthermore, the use of organic solvents poses risks to the internal environment of manned spacecraft cabins, while potential nanoparticle release during coating degradation could contaminate sensitive optical equipment. Therefore, future research must prioritize the development of scalable and environmentally friendly synthesis technologies tailored for space applications, along with comprehensive lifecycle assessments that cover both manufacturing and in-orbit performance.

### 7.2. Architectural and Agricultural Applications

POSS coatings are essential in both the construction and agricultural industries. In agriculture, they not only enhance light transmission, crucial for photosynthesis, but also shield plants from UV damage, making them ideal for greenhouse coverings [92,93]. For example, bio-based POSS-polyurethane nanocomposites—prepared through the liquefaction of starch in the presence of hydroxyl-containing POSS (POSS-OH) and polyethylene glycol, followed by crosslinking with polymethylene polyphenyl polysocyanate (PAPI)—form a nanocomposite coating with a nanoscale dispersion of POSS (100–500 nm), surface micro-protrusions, and an enhanced cross-linked structure. When used as urea coatings, these materials extend the nitrogen release period by one month, improve thermal stability and water resistance, promote plant growth, and enhance crop stress resistance through their UV protection properties. These materials are non-toxic to plants and can serve as eco-friendly alternatives to petroleum-based polymers [94]. The adoption of POSS in these high-volume sectors is constrained by economics. Bio-based POSS synthesis, while eco-friendly, involves complex liquefaction and cross-linking that increase production costs.

In the field of architecture, a POSS-modified wood coating achieves multifunctional integration of flame retardancy, antibacterial properties, and hydrophobicity through a chitosan/phytic acid layer-by-layer self-assembly process combined with a POSS-based flame retardant (Ag@bisDOPO-NH_2_-POSS), endowing the wood with self-extinguishing properties (LOI 30.9%), a 42.3% reduction in heat release rate, and simultaneous mold growth inhibition while maintaining a static contact angle >90°. This coating retains its hydrophobicity after 100 abrasion cycles [95], providing a highly durable and environmentally friendly solution for indoor/outdoor fire-resistant building materials (e.g., decorative panels, cabinets) and wooden components in humid environments (e.g., boardwalks, guardrails). Meanwhile, the advantages of POSS-based materials are also manifested in the field of demanding coatings. The copolymer synthesized through free radical polymerization of methacrylisobutyl POSS (MAPOSS) and glycidyl methacrylate (GMA) exhibits high transparency (>98% transmittance), strong adhesion (748.2 Pa), superior hydrophobicity (static contact angle: 108–112°), and controllable permeability. The nano-cage structure of POSS generates microporous channels within the coating, significantly surpassing the porosity of conventional epoxy resins which substantially enhances gas/liquid permeability [17]. These combined properties broaden its application prospects in decorative and high-performance architectural coatings.

### 7.3. Display Technology Application

POSS-based materials addressed core display trade-offs through its organic-inorganic hybrid structure. In flexible displays, cage-like octa(methacryloxypropyl) silsesquioxane (MA-POSS) and cage-like octa(3-mercaptopropyl) silsesquioxane (MP-POSS) are utilized. The silicon core of POSS provides essential hardness, while the thiol groups on the side chains impart high flexibility through chemical crosslinking. Leveraging the excellent compatibility and multifunctionality of these two POSS compounds, outstanding hardness (9H) and an exceptionally low elastic modulus (2.53 GPa) were achieved. When applied as a coating on polyethylene terephthalate (PET) film, it withstood 1000 cycles of steel wool abrasion while also enduring repeated bending for at least 1000 cycles (bending radius ~0.8 mm). This study offers a promising approach for developing surface protective coatings for next-generation foldable displays [9].

**Figure 12:**
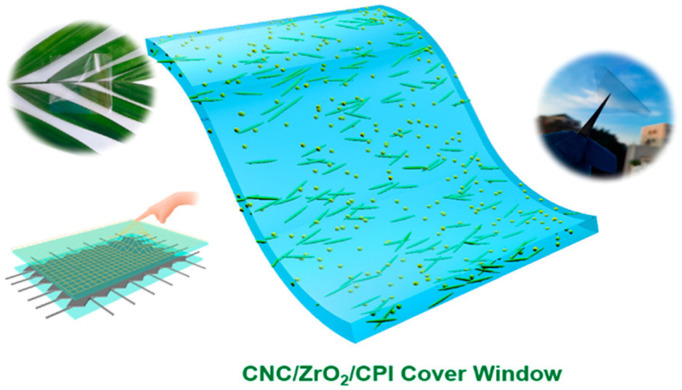
Schematic diagram of the CNC/ZrO_2_/CPI composite cover plate structure [98]. Copyright 2022 American Chemical Society.

In transparent displays, POSS-3, 6-dipyrene carbazole (POSS-DPCZ) hybrid material was used as an innovative material for the light-emitting layer. By constructing three-dimensional nanostructures of POSS and optimizing energy levels, a peak brightness of 8900 cd/m^2^ and stable blue light emission at 450 nm were achieved in three-layer OLEDs, with performance nearly twice that of pure DPCz devices [96]. Moreover, POSS capping layers (CPL) provide essential optical control: double-CPL structures enhance OLED brightness by 10% while reducing reflectivity and enable chromatic adaptation via RGB-transmissive subpixels [97]. For multifunctional encapsulation, POSS-polyimide composites mitigate persistent photoconductivity (PPC) in OLEDs [92], fluorinated MA_1.25_MP-g-F coatings offer oleophobic properties (hexadecane contact angle = 46°) [88], and POSS-nanocellulose hybrid cover windows achieve lightweight, foldable OLED encapsulation [93] (Figure 12). Scalable manufacturing and maintaining thick-film transmittance remain key challenges.

The superior properties of POSS coatings come with significant cost and processing challenges. High-purity, functional POSS monomers are substantially more expensive than conventional additives. Achieving nanoscale dispersion in polymer matrices is crucial for optical clarity but difficult to maintain in high-volume production. Integration into existing display manufacturing lines requires careful optimization of coating formulations and curing processes. Developing more cost-effective POSS synthesis routes and masterbatch pre-dispersions is essential for widespread adoption.

### 7.4. New Energy Applications

POSS has attracted considerable interest in the design of functional materials owing to its unique organic-inorganic hybrid nature. Its value is particularly demonstrated in the field of transparent coatings, as exemplified by the following applications:

As a transparent protective coating for photovoltaics, POSS-based coatings not only deliver excellent weather resistance, including protection against UV radiation and environmental erosion, along with self-cleaning capabilities (Section 6.2), but also maintain high light transmittance exceeding 90%. This combination effectively safeguards photovoltaic (PV) modules and significantly extends the service life of both ground-mounted power plants and building-integrated photovoltaics (BIPV) [99].

As a transparent functional layer for smart windows, when incorporated into electrochromic devices, POSS simultaneously provides high hardness, flexibility, and environmental stability without compromising optical clarity. By suppressing electrode degradation, it triples the switching cycle life and reduces energy consumption by 20%, thereby advancing the development of high-efficiency and energy-conserving smart windows [100].

### 7.5. Biological Applications

The combination of properties offered by POSS—optical transparency, tunable mechanical properties, and intrinsic biocompatibility—makes it highly valuable for transparent coatings in the biomedical field [101].

In the context of implantable devices, transparent coatings allow for in situ optical monitoring of the implantation site. For instance, the commercially implemented POSS-polycarbonate urea (POSS-PCU) nanocomposite has been used in implants such as synthetic tracheas [102].

For diagnostic and sensing platforms, POSS-based transparent coatings can serve as substrates for bioactive interfaces. In lab-on-a-chip systems and optical biosensors, the primary role of the coating is to maintain high light transmittance for fluorescence or colorimetric detection, while its surface must be biocompatible to suppress non-specific adsorption or to immobilize recognition molecules (e.g., antibodies, peptides) [103].

Furthermore, the ability of POSS to suppress molecular aggregation (see Section 4.2.1), combined with its potential for drug delivery, enables the creation of transparent drug-loaded coatings. Applying such a coating to devices like contact lenses or implantable optical sensors allows for the controlled release of therapeutic agents without compromising vision or signal transmission [104].

In summary, through its organic-inorganic hybrid nanostructure, POSS integrates optical transparency, mechanical enhancement, and biological function into a single material system. This establishes POSS-based materials as a key enabling platform for the development of transparent coatings for advanced biomedical devices, meeting the stringent requirements of applications at the intersection of optics and biology.

## 8. Discussion and Outlook

Polyhedral oligomeric silsesquioxane (POSS) transparent coatings represent a paradigm shift in material design, leveraging their unique organic-inorganic hybrid nanocage architecture to overcome longstanding limitations of conventional coatings. As summarized in this review, the strategic functionalization and advanced integration of POSS enable exceptional optical transparency, mechanical robustness, thermal stability, and tailored electronic properties. These attributes have propelled their application across diverse high-technology domains, from space-resistant spacecraft shielding to mechanically adaptive foldable displays and efficiency-enhanced energy devices. However, challenges remain that will possibly shape future research and potential applications.

Firstly, it is still important to elucidate the fundamental relationships between POSS cage architecture (particularly larger T_10_/T_12_ cages and emerging asymmetric/Janus-type structures) and material performance. While in the present literature T_8_-POSS systems have been extensively studied, larger cage systems offer higher free volume and altered packing dynamics that could enable ultralow dielectric constants (<2.0) while maintaining exceptional transparency and flexibility. For this point, computational modeling would be an effect tool to predict and verify how cage size, symmetry breaking, and functional group distribution affect.

Secondly, next-generation POSS coatings should evolve beyond single-function applications toward intelligent, responsive systems. Thus, the future of POSS-based transparent coatings lies in the improvement from single-property enhancement to comprehensive multifunctional design. By leveraging the programmable nature of POSS architecture through continued innovation in synthesis, functionalization, and integration techniques, researchers can create truly intelligent coatings such as self-healing capabilities for different damage types and adaptive properties that dynamically adjust their transparency [105], hydrophobicity, or electrical conductivity in response to environmental stimuli [9,106].

Finally, the practical application of POSS systems is heavily influenced by their commercial development status, which encompasses cost, structural modification capabilities, and scalability. The commercial landscape for POSS is currently characterized by a specialized supply chain. Companies like Hybrid Plastics Inc. have pioneered the commercialization of POSS, offering a diverse portfolio including functionalized cages (epoxy, methacrylate, amine), POSS-silanols, and even POSS-based monomers and polymers. This availability from commercial suppliers is a critical enabler for both research and early-stage industrial adoption. However, the high price of these materials, which often ranges from hundreds to several thousand US dollars per kilogram and is orders of magnitude greater than that of conventional polymers, remains a significant barrier to widespread adoption. This cost is intrinsically linked to the complex, multi-step synthesis and challenging purification processes required to achieve molecularly defined structures. The strategic importance of structural modification is evident in this commercial context: the selection of R groups directly determines the solubility, polymer matrix compatibility, and ultimate dispersion state of POSS, which in turn govern key performance properties. Therefore, future progress hinges not only on innovating more efficient and cost-effective synthetic routes but also on deepening the understanding of structure-property relationships to guide the rational design of POSS derivatives and their integration protocols for specific industrial applications.

## Figures and Tables

**Table 1:** Strategies for Tailoring the Properties of POSS or POSS-Filled Transparent Cover Plate Coatings.

Performance Property	Method/Strategy	Key Mechanism/Effect
Thermal Stability	Introduction of carborane cages into POSS framework	Mass loss below 10% after combustion at 1000 °C [14]
	Use of phenyl-functionalized POSS or longer/unsaturated side chains	Improved thermal stability and elasticity of polymer matrix [16]
	Cyclopentyl-substituted POSS (vs. isobutyl)	Thermally reinforced POSS domains enhance structural color stability [26]
Electronic Properties	Functionalization of POSS-T_8_ with electron-withdrawing/donating groups	Modulates HOMO/LUMO levels, bandgap, charge transport, and exciton binding energy [15]
	Insertion of molecules (e.g., N_2_) inside POSS cage	Lowers LUMO energy level [15]
	POSS as hole-blocking layer in perovskite LEDs	Deep HOMO level suppresses hole leakage, improves EQE [80]
	POSS-functionalized BNNTs in epoxy nanocomposites	Reduces dielectric constant and loss, enhances thermal conductivity [24]
Light Transmittance	Use of partially condensed POSS (vs. fully condensed)	Better dispersion in polymer matrix, higher transparency [21]
	Incorporation of TTB-modified POSS	UV-blocking while maintaining high visible-light transmittance [76]
	Low-crystallinity POSS with asymmetric substituents	Suppresses crystallization, forms optically transparent films [86]
	Dual curing (UV-thermal) with cycloaliphatic epoxy-POSS and acrylic resin A multi-point, highly cross-linked network was constructed through the efficient curing of a polyamine curing agent, tetraethylene-pentamine, with EP-POSS.	Highly transparent films with superior thermal stability [19] High transparency, low surface roughness, and efficient repellency against contaminants [77]
Mechanical Properties	In situ sol–gel synthesis of modified POSS with alkylsilanes	Reduces refractive index, improves thermal and mechanical properties of PMMA [22]
	POSS coating on carbon fibers via combined chemical-mechanical processing	Enhances interlaminar shear and impact resistance by 17–38% [78]
	Incorporation of diverse silsesquioxanes (SQ) into polyurethane acrylate (PUA)	Increases hardness and scratch resistance, reduces elastic modulus [23]
	Covalent grafting or chemical crosslinking of POSS	Prevents phase separation, enhances toughness, thermal stability, and network density [18]
Hydrophobicity	Copolymerization with 4M4F-POSS (four methacryloxy and four fluoroalkyl groups)	Enhances scratch resistance and hydrophobicity [27]
	Electrospinning of fluoroPOSS-PVDF-HFP nanocomposites	Forms transparent superhydrophobic coatings [83]
	Two-step dip-coating and hot-pressing with PDMS-ODA and fluorinated alumina nanoparticles	Retains transparency, imparts hydrophobicity on plastic substrates [28]
	Surface modification with fluorinated monomer (TFOA)	Imparts oil and water repellency without compromising other properties [9]
	Developing a fluorinated GPOSS-based coating with an inorganic/organic composite structure. Incorporation of poly(ethylene glycol) methyl ether methacry-late-functionalized POSS.	Significantly reduces the minimum de-icing pressure of the fluorinated modified coating [84]. Developing a highly hydrophilic coating, exhibiting a water contact angle of less than 10° [85].

**Table 2:** Comparison of Key Performance Parameters for Typical Transparent Coating Materials.

Performance Metric	Conventional Organic Coating (Reference Case)	Conventional Inorganic Coating (Reference Case)	POSS-Based Coating (Specific Example from This Review)
Optical Transmittance	Good: ~92% (Typical for clear polymers)	Excellent: >95% (e.g., Fused silica)	**>95% at 550 nm** achieved by using **partially condensed POSS** for superior dispersion in PMMA matrix, avoiding light scattering.
Hardness	Poor to Fair: 2B–3H (e.g., Soft PU/Acrylics)	Excellent but Brittle: 6H–9H (e.g., sol–gel SiO_2_)	**9H Pencil Hardness** achieved via a **crosslinked network of MA-POSS and MP-POSS**, providing inorganic-like rigidity.
Flexibility	Excellent: Can be folded	Very Poor: Cracks easily under strain	**Withstands > 1000 bending cycles (r ~0.8 mm)** due to the **flexible thiol-ene network** built from the same MA-POSS/MP-POSS system.
Thermal Stability	Poor: T_a_ or Td < 200 °C	Excellent: Td > 400 °C	**Significantly increased decomposition temperature** of silicone resin by incorporating **aminopropyl-POSS** forming reinforcing hydrogen bonds.
Environmental Resistance	Poor: Degrades under UV/AO	Excellent: Inert to UV/AO	**AO erosion rate reduced to 3.6% of pristine Kapton** using an **in situ-grown aminopropyl-POSS** adhesion layer under SiO_2_.
Surface Hydrophobicity	Moderate: ~95° (e.g., standard coatings)	Hydrophilic: <90°	**Superhydrophobic (Contact Angle > 110°)** achieved by **copolymerizing with 4M4F-POSS** in a UV-curable acrylate resin.
Key Limitation	Low durability, soft, poor thermal resistance	Brittle, poor adhesion, difficult to process	**Higher raw material cost; More complex synthesis and integration required.**

## Data Availability

No new data were created or analyzed in this study.
